# Adenosine Inhibits the Excitatory Synaptic Inputs to Basal Forebrain Cholinergic, GABAergic, and Parvalbumin Neurons in Mice

**DOI:** 10.3389/fneur.2013.00077

**Published:** 2013-06-20

**Authors:** Chun Yang, Serena Franciosi, Ritchie E. Brown

**Affiliations:** ^1^Laboratory of Neuroscience, Department of Psychiatry, VA Boston Healthcare System, Harvard Medical School, Brockton, MA, USA; ^2^Institute of Human Physiology II, University of Milan Medical School, Milan, Italy

**Keywords:** patch-clamp, adenosine A1 receptor, presynaptic modulation, sleep, transgenic mice

## Abstract

Coffee and tea contain the stimulants caffeine and theophylline. These compounds act as antagonists of adenosine receptors. Adenosine promotes sleep and its extracellular concentration rises in association with prolonged wakefulness, particularly in the basal forebrain (BF) region involved in activating the cerebral cortex. However, the effect of adenosine on identified BF neurons, especially non-cholinergic neurons, is incompletely understood. Here we used whole-cell patch-clamp recordings in mouse brain slices prepared from two validated transgenic mouse lines with fluorescent proteins expressed in GABAergic or parvalbumin (PV) neurons to determine the effect of adenosine. Whole-cell recordings were made from BF cholinergic neurons and from BF GABAergic and PV neurons with the size (>20 μm) and intrinsic membrane properties (prominent H-currents) corresponding to cortically projecting neurons. A brief (2 min) bath application of adenosine (100 μM) decreased the frequency but not the amplitude of spontaneous excitatory postsynaptic currents (EPSCs) in all groups of BF cholinergic, GABAergic, and PV neurons we recorded. In addition, adenosine decreased the frequency of miniature EPSCs in BF cholinergic neurons. Adenosine had no effect on the frequency of spontaneous inhibitory postsynaptic currents in cholinergic neurons or GABAergic neurons with large H-currents but reduced them in a group of GABAergic neurons with smaller H-currents. All effects of adenosine were blocked by a selective, adenosine A1 receptor antagonist, cyclopentyltheophylline (CPT, 1 μM). Adenosine had no postsynaptic effects. Taken together, our work suggests that adenosine promotes sleep by an A1 receptor-mediated inhibition of glutamatergic inputs to cortically projecting cholinergic and GABA/PV neurons. Conversely, caffeine and theophylline promote attentive wakefulness by inhibiting these A1 receptors in BF thereby promoting the high-frequency oscillations in the cortex required for attention and cognition.

## Introduction

Caffeine and theophylline are widely used psychostimulants that are commonly found in coffee, tea, and “energy” drinks (Sun et al., 2006; Pomeranz et al., 2013). These drugs act by inhibiting a group of highly conserved and widely expressed G-protein coupled proteins, adenosine receptors (Fredholm, 2010). These receptors’ endogenous agonist, adenosine, is considered a sleep homeostasis regulator (Basheer et al., 2004; Brown et al., 2012). Following sleep loss, there is a significant increase in the extracellular level of adenosine in basal forebrain (BF) and cortex (Porkka-Heiskanen et al., 1997, 2000; Kalinchuk et al., 2011) as well as in the mRNA and protein level of adenosine A1 receptors in BF (Basheer et al., 2001, 2007). This proportional relationship between the extracellular adenosine level and time spent in wakefulness is not brain wide but is limited to certain regions, most prominently the BF (Porkka-Heiskanen et al., 2000; Kalinchuk et al., 2011). However, the exact mechanisms by which adenosine modulates the BF neurons, especially the cortically projecting neurons, which in turn regulate the cortical activity, is still unclear.

The BF represents the final node of the ventral arm of the ascending reticular activating system (Moruzzi and Magoun, 1949), the chain of neural pathways arising from the brainstem which activates the cerebral cortex during wakefulness and rapid-eye-movement sleep (Semba, 2000; Jones, 2004). There are three major types of BF neurons classified according to their neurotransmitter phenotypes: cholinergic, GABAergic, and glutamatergic neurons (Gritti et al., 2006). Cholinergic neurons in the caudal BF including the magnocellular preoptic area (MCPO), horizontal limb of the diagonal band (HDB), ventral pallidum (VP), and substantia innominata (SI) are cortically projecting (Rye et al., 1984) and show maximum activity during waking and REM sleep (Manns et al., 2000a; Berntson et al., 2002), while the projection and discharge patterns during the sleep-wake cycle of BF GABAergic and glutamatergic neurons in these regions are more diverse (Manns et al., 2000b; Hassani et al., 2009). A significant proportion of GABAergic neurons project in parallel with cholinergic neurons to the cerebral cortex (Gritti et al., 1993; Henny and Jones, 2008) and show a wake/REM-on discharge pattern (Hassani et al., 2009). Thus, they may be involved in promoting cortical activation during wakefulness and REM sleep. Many of these cortically projecting GABAergic neurons express the calcium-binding protein parvalbumin (PV) and are fast firing (Gritti et al., 2003; McKenna et al., 2013). Consistent with a role in cortical activation, we recently found that optogenetic stimulation of BF PV neurons preferentially entrains cortical gamma band oscillations (Kim et al., 2011) which are important for attention and other cognitive functions during wakefulness.

Previous *in vivo* studies have shown that perfusion of adenosine or an adenosine transporter blocker into the rat BF decreases wakefulness (Porkka-Heiskanen et al., 1997; Basheer et al., 1999). Patch-clamp studies on *rat* brain slices showed that extracellular adenosine inhibited caudal BF cholinergic neurons mediated via A1 receptors and decreased the firing of neighboring non-cholinergic neurons (Arrigoni et al., 2006). Furthermore, a recent study in mouse brain slices suggested that adenosine also inhibits the excitatory glutamatergic inputs to BF cholinergic neurons via A1 receptors (Hawryluk et al., 2012). While much is known about the adenosine modulation of the BF cholinergic system, less is known about its effect on the cortically projecting BF GABAergic/PV neurons due to difficulties in positively identifying these neurons. Given that cortically projecting GABAergic/PV neurons likely have similar functions as cholinergic neurons in promoting cortical activation, we hypothesized that in mouse, adenosine would inhibit wake-active/promoting cortically projecting BF neurons via A1 receptor-mediated inhibition of excitatory synaptic inputs.

We recently validated the use of GAD67-GFP knock-in mice and PV-tomato mice as effective models to identify BF GABAergic and PV neurons respectively. Furthermore, we showed that large (>20 μm long diameter) GABAergic neurons with prominent hyperpolarization-activated inward currents (*I*_h_) in MCPO/HDB regions were cortically projecting (McKenna et al., 2013). Taking advantage of these genetically modified animal models, in the current study, we re-examined the presynaptic and postsynaptic modulation of adenosine on BF cholinergic neurons and for the first time tested the adenosine effects on identified BF cortically projecting GABAergic and PV neurons by whole-cell patch-clamp. Some of this work has been reported in abstract form Yang et al. (2011).

## Materials and Methods

### Animals

All experiments conformed to U.S. Veterans Administration, Harvard University, and U.S. National Institutes of Health guidelines and were reviewed by the institutional animal care and use committee (IACUC) of the VA Boston Healthcare System. Experimental male and female GAD67-GFP knock-in animals were obtained by crossing male heterozygous GAD67-GFP knock-in mice (Swiss-Webster background) with wild-type female Swiss-Webster mice (Charles River, Wilmington, MA, USA). GFP-positive animals were phenotyped under a fluorescent microscope within 3 days after birth. GAD67-GFP knock-in animals have similar sleep-wake behavior and cortical rhythms as wild-type animals (Chen et al., 2010; McNally et al., 2011). PV-tomato male and female animals were obtained by crossing female homozygous Cre-tomato mice (Strain 007905, Jackson lab) with male homozygous PV-Cre mice (Strain 008069, Jackson lab). The selective expression of these fluorescent proteins in GABAergic and PV neurons were validated in our previous study (McKenna et al., 2013). The intrinsic membrane properties of identified cortically projecting BF GABAergic and PV neurons as well as those of BF cholinergic neurons using these two transgenic models was also reported in that study and are used here for identification purposes. Mice were housed under constant temperature and a 12:12 light:dark cycle (7a.m.:7p.m.), with food and water available *ad libitum*.

### Slice preparation

Slices from young mice (12–22 days) were used for most experiments unless otherwise specified. Mice were deeply anesthetized with isoflurane and then decapitated. Coronal BF slices (300 μm thickness) were cut between 0.26 and −0.22 mm with respect to Bregma rostrocaudally in ice-cold sucrose solution (in mM: 208.6 sucrose, 1.8 KCl, 25.6 NaHCO_3_, 1.2 KH_2_PO_4_, 0.6 CaCl_2_, 3.3 MgSO_4_, 10 glucose, saturated with 95% O_2_/5% CO_2_). After slicing they were placed into ACSF (in mM: 124 NaCl, 1.8 KCl, 25.6 NaHCO_3_, 1.2 KH_2_PO_4_, 2 CaCl_2_, 1.3 MgSO_4_ and 10 glucose, 300 mOsm, saturated with 95% O_2_/5% CO_2_) for>1 h at room temperature before being transferred to the recording chamber and superfused with warmed ACSF (32°C) at 2–3 ml/min.

To test the effect of adenosine in *adult* BF cholinergic neurons, GAD67-GFP mice of 1.5–3 month old were used. Mice were deeply anesthetized by intra-peritoneal injection of pentobarbital and then transcardially perfused with 25 ml of ice-cold *N*-methyl-d-glucamine (NMDG)-solution (in mM): 92 NMDG, 2.5 KCl, 1.25 NaH_2_PO_4_, 30 NaHCO_3_, 20 HEPES, 25 glucose, 2 thiourea, 5 Na-ascorbate, 3 Na-pyruvate, 0.5 CaCl_2_, and 10 MgSO_4_, pH 7.3, 300 mOsm. Mice were then decapitated and the brains were removed into the ice-cold NMDG-solution for 1–2 min. The brains were sectioned at 300 μM thickness in ice-cold NMDG-solution. Coronal slices containing BF were incubated for 15 min at 35°C in NMDG-solution and then transferred to a modified ACSF solution (containing in mM, 119 NaCl, 2.5 KCl, 1.25 NaH_2_PO_4_, 26 NaHCO_3_, 12.5 glucose, 2 CaCl_2_, 2 MgSO_4_, 2 mM thiourea, 5 Na-ascorbate, and Na-pyruvate, pH 7.3, 300 mOsm.) for at least 1 h at room temperature. All recordings were performed in regular ACSF.

Due to the late postnatal developmental expression pattern of PV (de Lecea et al., 1995; Lohmann and Friauf, 1996; Collin et al., 2005), PV-tomato animals from 14 to 60 days old were used for this study. The slice preparation procedures were the same as for GAD67-GFP pups and adults.

### Whole-cell patch-clamp recordings

**Figure 1:**
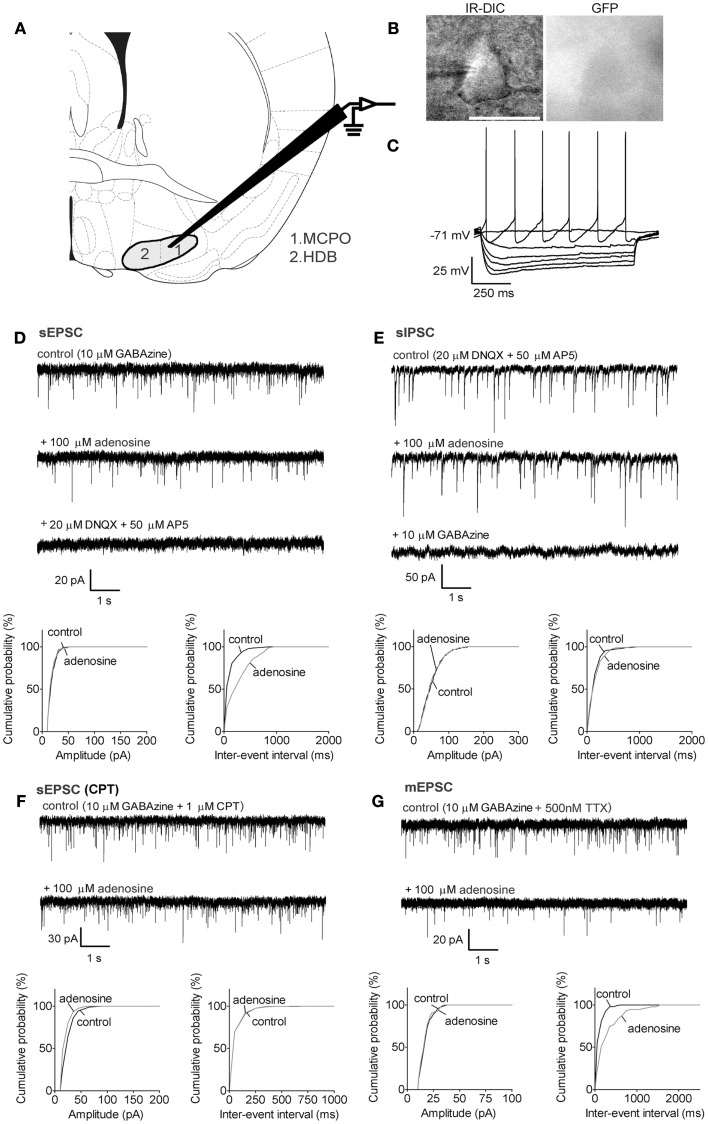
**Adenosine decreases the frequency of excitatory synaptic inputs onto BF cholinergic neurons via adenosine A1 receptors but does not affect inhibitory inputs**. **(A)** Schematic (adapted from mouse atlas of Franklin and Paxinos, 2008; AP 0.14 mm) showing the location of the BF recording sites in the magnocellular preoptic area (1. MCPO) and horizontal limb of the diagonal band (2. HDB). **(B)** Infrared-differential interference microscopy (IR-DIC) and black-and-white fluorescent images of a typical cholinergic neuron. Scale bar: 25 μm. Cholinergic neurons were distinguished from GABAergic neurons by their GFP-negative pattern under fluorescent illumination and identified by their typical intrinsic membrane properties during current pulses **(C)** −80 to +16 pA, −16 pA interval. Cholinergic neurons were silent at rest and exhibited slow firing and large afterhyperpolarizations when depolarized. In our previous study (McKenna et al., 2013) neurons with these properties were all identified as cholinergic by *post hoc* immunohistochemistry. **(D)** Adenosine reduced the frequency but not the amplitude of spontaneous excitatory postsynaptic currents (sEPSC). The GABA_A_ receptor antagonist, GABAzine, was used to isolate excitatory events. **(E)** Adenosine did not affect the frequency or amplitude of spontaneous inhibitory postsynaptic currents (sIPSC). The ionotropic glutamate receptor antagonists, DNQX and AP5 were used to isolate inhibitory synaptic events. A KCl-based intracellular solution was used to enhance the driving force for chloride. This shifted the reversal potential in the depolarizing direction, thus the recorded currents are inward going. **(F)** The adenosine A1 receptor antagonist, 1 μM CPT, blocked the effect of adenosine on sEPSCs. **(G)** Adenosine reduced the frequency of miniature excitatory postsynaptic currents (mEPSCs), recorded in the presence of tetrodotoxin (TTX) and GABAzine. **(D–G)** *Top traces:* 10 s-long representative traces from one neuron under each recording condition. Holding potential was −70 mV. *Bottom graphs:* cumulative probability histograms of amplitude (left) and inter-event interval (right) of 1 min recordings from the same neuron. Distributions were compared using the Kolmogorov–Smirnov test.

For cholinergic and GABAergic neurons, electrophysiological recordings were made from somata of neurons in the HDB and MCPO of BF (Figure 1A) where the majority of large, cortically projecting cholinergic and GABAergic neurons were reported (Jones, 2004; McKenna et al., 2013). For PV neurons, neurons in VP were also included. Their intrinsic properties and responses to adenosine were similar to those in MCPO/HDB and therefore the results have been pooled. Putative cortically projecting BF neurons were selected for recording based on their morphology, intrinsic membrane properties, and the fluorescent protein expression (McKenna et al., 2013).

**Figure 2:**
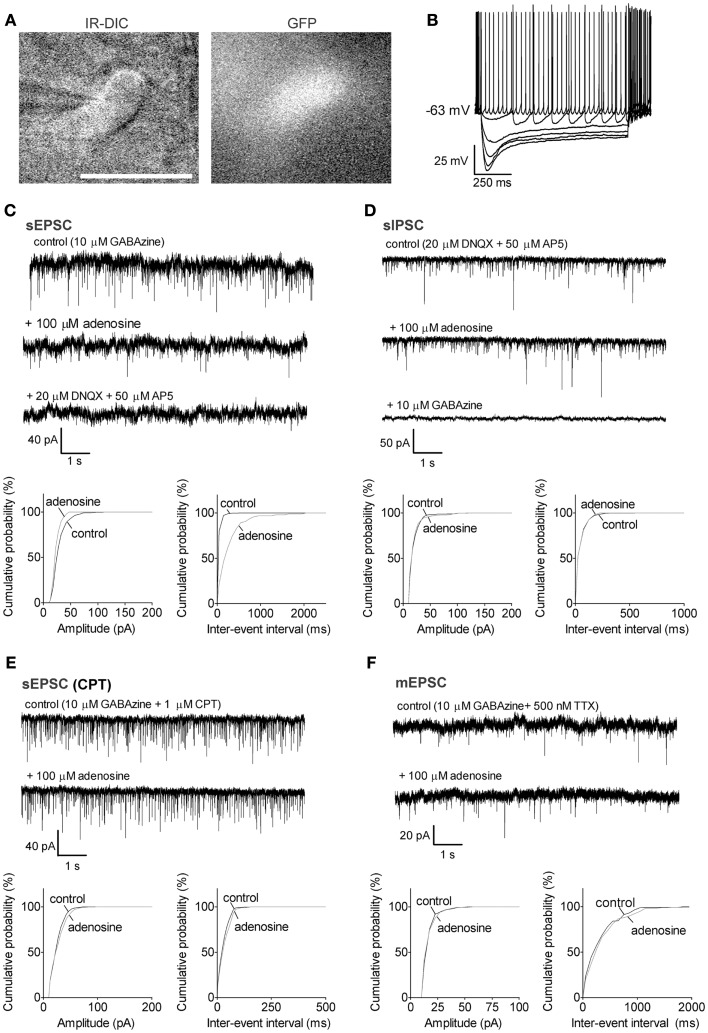
**Adenosine significantly decreased the frequency of sEPSCs via A1 receptors in GABAergic neurons with a large *I*_h_**. **(A)** Infrared-differential interference microscopy (IR-DIC) and black-and-white fluorescent images of a typical GABAergic neuron with a large *I*_h_. Scale bar: 25 μm. GABAergic neurons were distinguished from cholinergic neurons by their GFP-positive pattern under fluorescent illumination and identified by their typical intrinsic membrane properties during current pulses **(B)** −500 to 0, −100 pA interval. GABAergic neurons with a large *I*_h_ were spontaneously active, and exhibited large depolarizing sags during hyperpolarizing current pulses and small afterhyperpolarizations when depolarized. **(C)** Adenosine reduced the frequency but not the amplitude of spontaneous excitatory postsynaptic currents (sEPSC). The GABA_A_ receptor antagonist, GABAzine, was used to isolate excitatory events. Holding potential was −60 mV. **(D)** Adenosine did not affect the frequency or amplitude of spontaneous inhibitory postsynaptic currents (sIPSC). The ionotropic glutamate receptor antagonists, DNQX and AP5 were used to isolate inhibitory synaptic events. A KCl-based intracellular solution and a holding potential of −90 mV were used to enhance the driving force for chloride. KCl-based intracellular solution shifted the reversal potential in the depolarizing direction, thus the recorded currents are inward going. **(E)** The adenosine A1 receptor antagonist, 1 μM CPT, blocked the effect of adenosine on sEPSCs. **(F)** Adenosine did not affect the frequency or amplitude of miniature excitatory postsynaptic currents (mEPSCs), recorded in the presence of tetrodotoxin (TTX) and GABAzine. **(C–F)** *Top traces:* 10 s-long representative traces from one neuron under each recording condition. *Bottom graphs:* cumulative probability histograms of amplitude (left) and inter-event interval (right) of 1 min recordings from the same neuron. Distributions were compared using the Kolmogorov–Smirnov test.

**Figure 3:**
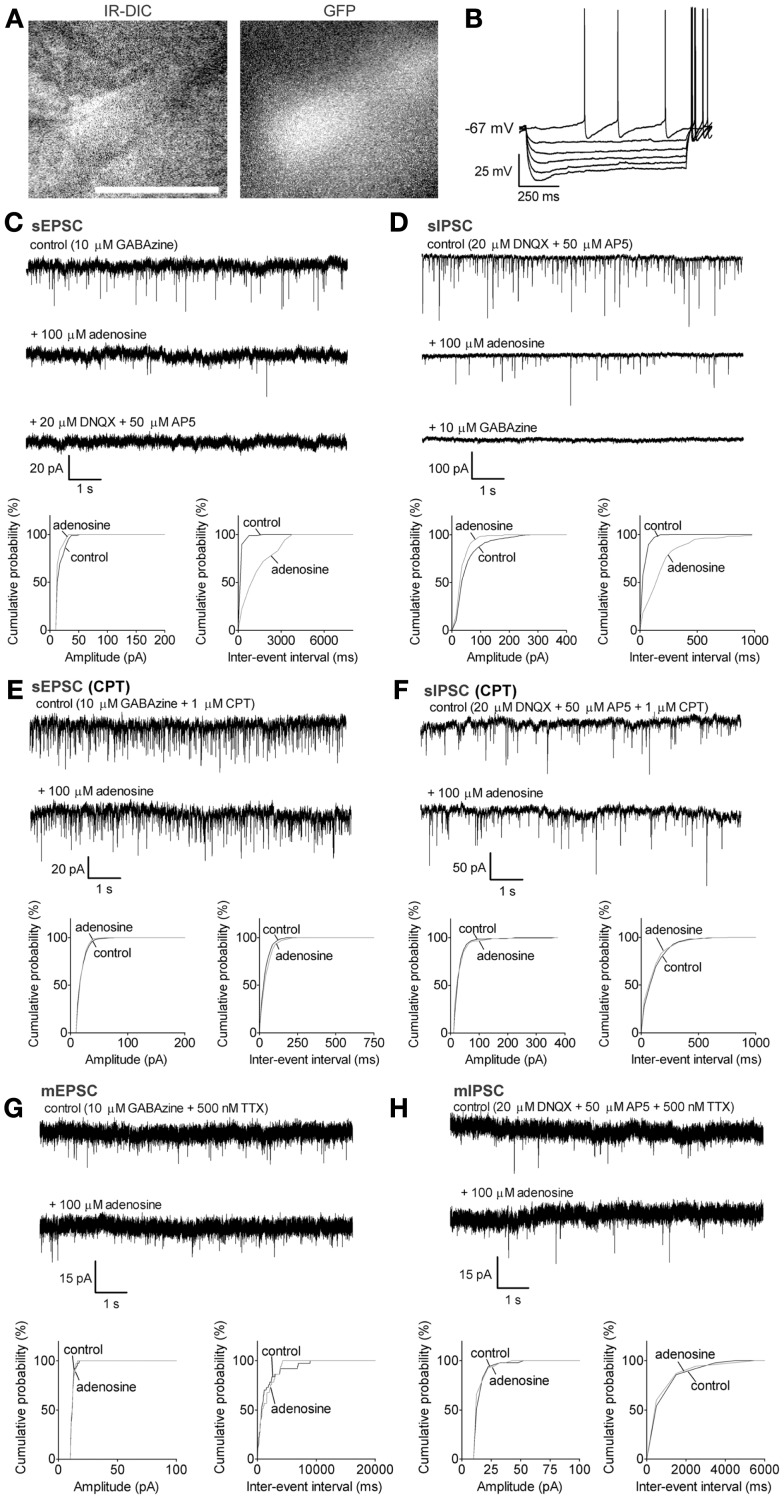
**Adenosine significantly decreases the frequency of sEPSC and sIPSC via A1 receptors GABAergic neurons with a small *I*_h_**. **(A)** Infrared-differential interference microscopy (IR-DIC) and black-and-white fluorescent images of a typical GABAergic neuron with a small *I*_h_. Scale bar: 25 μm. GABAergic neurons were distinguished from cholinergic neurons by their GFP-positive pattern under fluorescent illumination and identified by their typical intrinsic membrane properties during current pulses **(B)** −300 to 0, −60 pA interval. GABAergic neurons with a small *I*_h_ were spontaneously active, and exhibited small depolarizing sags during hyperpolarizing current pulses and small afterhyperpolarizations when depolarized. **(C)** Adenosine reduced the frequency but not the amplitude of spontaneous excitatory postsynaptic currents (sEPSC). The GABA_A_ receptor antagonist, GABAzine, was used to isolate excitatory events. Holding potential was −60 mV. **(D)** Adenosine reduced the frequency but not the amplitude of spontaneous inhibitory postsynaptic currents (sIPSC). The ionotropic glutamate receptor antagonists, DNQX and AP5 were used to isolate inhibitory synaptic events. A KCl-based intracellular solution and a holding potential of −90 mV were used to enhance the driving force for chloride. KCl-based intracellular solution shifted the reversal potential in the depolarizing direction, thus the recorded currents are inward going. **(E)** The adenosine A1 receptor antagonist, 1 μM CPT, blocked the effect of adenosine on sEPSCs. **(F)** CPT blocked the effect of adenosine on sIPSCs. **(G)** Adenosine did not affect the frequency or amplitude of miniature excitatory postsynaptic currents (mEPSCs), recorded in the presence of tetrodotoxin (TTX) and GABAzine. **(H)** Adenosine did not affect the frequency or amplitude of miniature inhibitory postsynaptic currents (mIPSCs), recorded in the presence of TTX, DNQX, and AP5. **(C–H)** *Top traces:* 10 s-long representative traces from one neuron under each recording condition. *Bottom graphs:* cumulative probability histograms of amplitude (left) and inter-event interval (right) of 1 min recordings from the same neuron. Distributions were compared using the Kolmogorov–Smirnov test.

In GAD67-GFP animals, putative cholinergic neurons were GFP-negative with a size larger than 20 μm (Figure 1B, long diameter 22.0 ± 0.5 μm, *n* = 32), were not normally spontaneously active (Figure 1C, spontaneous firing frequency: 3.3 ± 0.8 Hz, *n* = 8/32; resting membrane potential: −73.2 ± 1.1 mV, *n* = 32) and exhibited a large afterhyperpolarization (−31.2 ± 0.9 mV). GABAergic neurons of interest were GFP-positive with a size larger than 20 μm (Figures 2A and 3A, 22.5 ± 0.5 μm at long diameter, *n* = 72) and often showed spontaneous action potential discharge (Figures 2B and 3B, spontaneous firing frequency: 12.2 ± 1.3 Hz; resting membrane potential: −66.8 ± 0.5 mV) and a smaller afterhyperpolarization (−16.3 ± 0.7 mV). In PV-tomato animals, fluorescent PV neurons showed similar morphology and electrophysiological patterns as cortically projecting GABAergic neurons (long diameter: 23.4 ± 1.3 μm; spontaneous firing: 20 ± 12.6 Hz; resting membrane potential: −8.3 ± 3.5 mV; amplitude of afterhyperpolarization: −15.9 ± 2.4 mV; *n* = 5) but had action potentials with shorter duration than those of cholinergic and GABAergic neurons (action potential half width: 0.26 ± 0.03 ms for PV neurons; 0.85 ± 0.04 ms and 0.76 ± 0.03 ms for cholinergic and GABAergic neurons).

Neurons were photographed prior to recording using a Hamamatsu ORCA-ER CCD camera (Hamamatsu Corporation, Middlesex, NJ, USA). Fluorescent neurons were observed with Zeiss filters (GFP: filter set 38, excitation filter 470/40, and emission filter 525/50; Tomato: filter set 45, excitation filter 560/40, and emission filter 630/75). Long-axis cell diameter was measured from these images and calibrated using a standard 25 μm grid.

All recordings were made using a Multiclamp 700B amplifier and pClamp 9.0 software (Molecular Devices, LLC, Sunnyvale, CA, USA). Patch pipettes (3–6 MΩ) were filled with intracellular solution containing in mM: 130 K-gluconate, 5 NaCl, 2 MgCl_2_, 10 HEPES, 0.1 EGTA, 2 Na_2_ATP, 0.5 NaGTP, 4 MgATP, 1 spermine (pH 7.25 with KOH, 280 mOsm) (Sigma, St. Louis, MO, USA). Membrane potential measurements were adjusted for liquid junction potentials between the pipette and bath solution. Bridge balance was adjusted after gaining access to the whole-cell and maintained throughout the experiment. Recordings were accepted if action potentials were overshooting and electrode resistance was less than 20 MΩ and changed by less than 10% during the experiment. Continuous recordings of synaptic currents were made using a MiniDigi 1A system and Axoscope 9.2 software (Molecular Devices, LLC, Sunnyvale, CA, USA) with a sampling frequency of 20 kHz.

### Testing the postsynaptic and presynaptic effect of adenosine

In order to rapidly obtain a maximal effect, adenosine was bath applied at a concentration of 100 μM, as in other *in vitro* studies (Arrigoni et al., 2006; Hawryluk et al., 2012). Since equilibration of solutions between the bath and within the slice takes ∼10–15 min the actual adenosine concentration in the vicinity of the recorded neurons is likely to have been considerably lower. To test the postsynaptic effect of adenosine, neurons were pre-incubated in 500 nM tetrodotoxin (TTX) (Abcam, Cambridge, MA, USA) for 5 min and then tested in response to 2 min bath application of adenosine under current clamp (*I*_hold_ = 0). Patch pipettes were filled with regular intracellular solution.

To test the presynaptic effect of adenosine, spontaneous postsynaptic currents (sPSCs) were recorded without TTX while miniature postsynaptic currents (mPSCs) were recorded in the presence of 500 nM TTX. Excitatory postsynaptic currents (EPSCs) were recorded with 10 μM GABAzine (Abcam, Cambridge, MA, USA) in the bath to block GABA_A_ receptor-mediated currents and at a holding potential close to the resting membrane potentials of these neurons (cholinergic neurons at −70 mV and GABAergic neurons at −60 mV). At the end of EPSCs recordings, 20 μM 6,7-dinitroquinoxaline-2,3-dione (DNQX) (Abcam, Cambridge, MA, USA) and 50 μM (2R)-amino-5-phosphonovaleric acid (AP5) (Abcam, Cambridge, MA, USA) which block glutamatergic AMPA and NMDA receptor-mediated currents respectively, were applied to the bath to confirm those excitatory inputs were glutamatergic. Inhibitory postsynaptic currents (IPSCs) were recorded in the presence of 20 μM DNQX and 50 μM AP5. The holding potential was −70 mV for cholinergic neurons and −90 mV for GABAergic neurons. In addition, for recording of IPSCs, K-gluconate was replaced with KCl in the patch pipettes in order to enhance the driving force for chloride entry and thus the resolution of GABA_A_ receptor-mediated events. At the end of IPSCs recordings, 10 μM GABAzine was applied to the bath to confirm those inhibitory inputs were GABAergic. All receptor antagonists including the adenosine receptor antagonist, cyclopentyltheophylline (CPT) (Sigma, St. Louis, MO, USA) were bath applied for 5 min before the adenosine response was tested. Adenosine (Sigma, St. Louis, MO, USA) was bath applied for ∼3 min. A 1-min period immediately prior to adenosine application and a 1-min period after 2 min application of adenosine were used for statistical analysis with Igor software (WaveMetrics, Inc., Portland, OR, USA). Only well resolved events with amplitudes>10 pA were analyzed. The baseline current during postsynaptic current recordings was measured by lowpass filtering the trace (Bessel, 8-pole) at 10 Hz in pClamp to eliminate the synaptic currents.

### Data analysis and statistics

The morphological and electrophysiological parameters of BF neurons were analyzed using the same methods as previously reported (McKenna et al., 2013). Data were presented as mean ± standard error of the mean (SEM). For data with normal distribution, paired *t* test and unpaired *t* test were used for statistical analysis of significance. Kolmogorov–Smirnov two-sample test was used to test significant difference for cumulative probabilities. Statistical analysis utilized GraphPad Prism 4 (GraphPad Software, Inc., La Jolla, CA, USA), and differences were considered significant when *p* < 0.05.

## Results

### Effect of adenosine on putative cholinergic neurons

#### Adenosine presynaptically inhibits glutamatergic inputs to cholinergic neurons via A1 receptors but does not affect GABAergic inputs

Putative cholinergic neurons in MCPO/HDB (Figure 1A) were identified as GFP-negative neurons (Figure 1B) with intrinsic membrane properties similar to those previously reported for cholinergic neurons by us (McKenna et al., 2013) and others (e.g., Hawryluk et al., 2012) (see Materials and Methods for the properties of the neurons used in the current study).

Bath application of adenosine (100 μM) reduced the frequency of sEPSCs from 11.1 ± 2.4 to 4.5 ± 1.2 Hz (*p* = 0.0121, paired *t* test, *n* = 5) but there was no significant change in the amplitude (control vs. adenosine: 23.6 ± 2.4 vs. 20.6 ± 1.0 pA, *p* = 0.1299, paired *t* test, Figure 1D), suggesting adenosine modulation of excitatory inputs was via a presynaptic mechanism. The baseline current was also significantly changed after adenosine incubation (from −51.0 ± 8.9 to −41.7 ± 7.7 pA, *p* = 0.009, paired *t* test), which resulted in an outward current of 9.4 ± 2.0 pA in response to adenosine. Adenosine did not change the frequency or amplitude of sIPSCs recorded using KCl-filled electrodes in the presence of 20 μM DNQX and 50 μM AP5 (frequency: 6.5 ± 1.9 vs. 6.0 ± 1.8 Hz, *p* = 0.8024; amplitude: 50.0 ± 5.8 vs. 44.9 ± 5.8 pA, *p* = 0.1058, paired *t* test, *n* = 8, Figure 1E).

Presynaptic inhibitory effects of adenosine on glutamatergic synaptic transmission are normally mediated via adenosine A1 receptors (Greene and Haas, 1991; Flagmeyer et al., 1997; Brown et al., 2012). In our experiments, adding the A1 receptor antagonist, 1 μM CPT, 5 min prior to the application of adenosine, completely blocked the adenosine effects on sEPSCs (frequency: 4.8 ± 1.7 vs. 3.9 ± 1.6 Hz in adenosine, *p* = 0.1147; amplitude: 22.3 ± 2.7 vs. 21.7 ± 2.9 pA in adenosine, *p* = 0.6449, paired *t* test, *n* = 6, Figure 1F) as well as on baseline current (CPT vs. CPT + adenosine: −61.2 ± 8.0 vs. −62.9 ± 7.4 pA, *p* = 0.3852, paired *t* test), suggesting that adenosine inhibited the excitatory inputs to cholinergic neurons by activating A1 receptors. The sEPSC frequency or amplitude in CPT was not significantly different from those recorded without CPT (*p* = 0.0528 for frequency, *p* = 0.7455 for amplitude, unpaired *t* test) suggesting a lack of endogenous adenosine tone on cholinergic neurons under our recording conditions.

Spontaneous postsynaptic currents can be generated by action potential-dependent release of neurotransmitter due to firing of local neurons in the slice or due to spontaneous, action potential-independent release from synaptic terminals. To assess if adenosine acts directly on the presynaptic terminals we recorded action potential-independent postsynaptic currents (miniature PSCs) in the presence of TTX (500 nM). Adenosine significantly decreased the frequency of mEPSCs (4.4 ± 1.2 vs. 2.6 ± 0.8 Hz in adenosine, *p* = 0.0481, paired *t* test, *n* = 7, Figure 1G) but had no effect on the amplitude (18.7 ± 1.8 vs. 18.3 ± 1.6 pA in adenosine, *p* = 0.4972, paired *t* test). The decrease in frequency of mEPSCs with adenosine incubation was 40.0 ± 7.3%, which was significantly smaller than the decrease of sEPSC frequency in adenosine (60.9 ± 5.1% decrease) (*p* = 0.0452, unpaired *t* test), suggesting that adenosine inhibits action potentials in local glutamatergic neurons as well as directly inhibiting transmitter release from presynaptic terminals. In the presence of TTX, there was no significant change in the baseline current after adenosine incubation (TTX vs. TTX + adenosine: −50.5 ± 9.6 vs. −47.3 ± 8.4 pA, *p* = 0.1230, paired *t* test). The lack of adenosine effect on baseline current in the presence of TTX suggests that the effect of adenosine on baseline current in control conditions (no TTX) is most likely due to presynaptic inhibition of a tonic glutamatergic tone which normally depolarizes cholinergic neurons.

It has been reported that adenosine had a postsynaptic inhibition on BF cholinergic neurons in rat pups (Arrigoni et al., 2006). Our voltage-clamp recordings suggested this was not the case in the mouse. To further confirm our findings, we applied 100 μM adenosine to the bath in the presence of 500 nM TTX and recorded the voltage response under current clamp. We found that none of the cholinergic neurons in pups or adults had a direct response to adenosine (mean ± SEM voltage changes: −0.59 ± 1.87 mV, *p* = 0.7674, *n* = 5 from pups; −0.94 ± 0.84 mV, *p* = 0.3282, *n* = 5 from adults). These data were consistent with our voltage-clamp recordings where no change in baseline current after adenosine application was observed in the presence of TTX.

### Adenosine effects on putative cortically projecting GABAergic neurons

Previous studies in the rat have shown that a subset of GABAergic neurons, including those expressing PV, project to the cortex (Gritti et al., 2003). Our previous work using retrograde tracing in mouse brain suggested that large GABAergic neurons in caudal BF project directly to the prefrontal cortex (McKenna et al., 2013). Thus, here we only focused on the large GABAergic neurons (>20 μm) which are likely to be cortically projecting. In our previous study, we subdivided the large GABAergic neurons in MCPO/HDB into two groups based on the size and kinetics of their depolarizing sag during hyperpolarizing current pulses (McKenna et al., 2013). We continued to use the same criterion for the current study and investigated the adenosine effect on each group. The size and intrinsic membrane properties of the GABAergic neurons were presented in the Section “Materials and Methods.” GABAergic neurons which had a bi-exponential curve (τ_fast_ = 72.4 ± 4.8 ms, τ_slow_ = 895 ± 241 ms, *n* = 29) which best fit the depolarizing sag during a step to −110 mV and often showed a large sag (50.9 ± 1.8%) were defined as large *I*_h_ GABAergic neurons (Figure 2B), while neurons with a monoexponential curve best fit (τ = 353 ± 80 ms, *n* = 43) and a significantly smaller sag (27.2 ± 1.8%, *p* < 0.0001, unpaired *t* test) were defined as small *I*_h_ GABAergic neurons (Figure 3B). There was no difference in morphology between these two groups.

#### Adenosine inhibits the action potential-dependent excitatory inputs to large *I*_h_ GABAergic neurons via A1 receptors and a presynaptic mechanism, but has no effect on their inhibitory inputs

In large *I*_h_ GABAergic neurons, adenosine significantly decreased the frequency of sEPSCs from 10.4 ± 2.7 to 3.7 ± 2.4 Hz (67.5 ± 12.9% decrease, *p* = 0.0218, paired *t* test, *n* = 6, Figure 2C) with no change in the amplitude (20.5 ± 2.5 vs. 19.4 ± 3.2 pA in adenosine, *p* = 0.7011). All sEPSCs were completely eliminated with DNQX and AP5 added to the bath. These results suggested that adenosine inhibited the glutamatergic excitatory inputs to large *I*_h_ GABAergic neurons by a presynaptic mechanism. A significant change in baseline current was also observed in those large *I*_h_ GABAergic neurons with adenosine incubation (from −211.2 ± 73.7 to −193.0 ± 70.0 pA, *p* = 0.0321, paired *t* test), which resulted in an outward current of 18.2 ± 6.2 pA in response to adenosine. Similar to cholinergic neurons, adenosine did not affect the sIPSCs of large *I*_h_ GABAergic neurons (frequency: 7.6 ± 2.7 vs. 7.9 ± 2.7 Hz, *p* = 0.2094; amplitude: 28.8 ± 4.6 vs. 28.7 ± 4.8 pA, *p* = 0.9416, paired *t* test; *n* = 5, Figure 2D). CPT completely blocked the adenosine effects on sEPSCs (frequency: 13.7 ± 4.3 vs. 14.8 ± 4.0 Hz, *p* = 0.7045; amplitude: 19.3 ± 1.9 vs. 19.9 ± 2.5 pA, *p* = 0.6447, paired *t* test, *n* = 5, Figure 2E) and the effect on baseline current (CPT vs. CPT + adenosine: −174.0 ± 58.5 vs. −176.9 ± 58.2 pA, *p* = 0.5482, paired *t* test), suggesting that adenosine effects were mediated via A1 receptors. There was no significant difference between sEPSCs recorded in regular ACSF and those recorded in CPT (frequency: *p* = 0.5066, unpaired *t* test; amplitude: *p* = 0.7087, unpaired *t* test). Unlike cholinergic neurons, adenosine did not affect the mEPSC of large *I*_h_ GABAergic neurons (frequency: 5.2 ± 3.2 vs. 4.8 ± 3.5 Hz, *p* = 0.3942; amplitude: 18.5 ± 2.1 vs. 18.0 ± 1.6 pA, *p* = 0.4159, paired *t* test; *n* = 5, Figure 2F). As with cholinergic neurons, the baseline current in the presence of TTX was not affected by adenosine (TTX vs. TTX + adenosine, −54.4 ± 43.1 vs. −50.8 ± 41.6 pA, *p* = 0.3723, paired *t* test).

#### Adenosine inhibits the action potential-dependent excitatory and inhibitory inputs to small *I*_h_ GABAergic neurons via A1 receptors and a presynaptic mechanism

By using similar approaches as for large *I*_h_ GABAergic neurons, we investigated the adenosine effects on the synaptic inputs to small *I*_h_ GABAergic neurons. The frequency of sEPSCs was significantly decreased from 7.5 ± 2.2 to 1.9 ± 1.2 Hz with adenosine incubation (78.9 ± 6.1% decrease, *p* = 0.0102, paired *t* test, *n* = 8, Figure 3C). There was no significant change in the amplitude (20.7 ± 2.0 vs. 17.4 ± 0.9 pA, *p* = 0.0590, paired *t* test) or in the baseline current (−121.8 ± 31.5 vs. −110.3 ± 29.6 pA, *p* = 0.0701, paired *t* test), suggesting there was no postsynaptic effect. Unlike cholinergic and large *I*_h_ GABAergic neurons, the sIPSCs of small *I*_h_ GABAergic neurons were also affected by adenosine. The frequency of sIPSCs in regular ACSF was 16.6 ± 4.2 Hz and decreased to 11.5 ± 4.0 Hz in the presence of adenosine (37.1 ± 11.1% decrease, *p* = 0.0277, paired *t* test; *n* = 8, Figure 3D). There was no significant change in the amplitude (amplitude: 56.0 ± 12.2 vs. 42.3 ± 7.9 pA, *p* = 0.0960, paired *t* test).

Cyclopentyltheophylline completely blocked the adenosine effects on sEPSCs (frequency: 33.7 ± 12.7 vs. 34.8 ± 12.9 Hz, *p* = 0.9013; amplitude: 21.1 ± 2.2 vs. 22.6 ± 2.6 pA, *p* = 0.5605, paired *t* test, *n* = 5, Figure 3E). The frequency of sEPSCs recorded in CPT was significantly larger than that recorded in regular ACSF (frequency: 33.7 ± 12.7 Hz in CPT vs. 7.5 ± 2.2 Hz in ACSF, *p* = 0.0254; amplitude: 21.1 ± 2.2 pA in CPT vs. 20.7 ± 2.0 pA in ACSF, *p* = 0.9033; unpaired *t* test), suggesting the existence of an endogenous adenosine inhibition of small *I*_h_ GABAergic neurons. The adenosine effect on sIPSCs was also blocked by CPT (frequency: 14.6 ± 2.4 vs. 14.0 ± 2.6 Hz, *p* = 0.6511; amplitude: 80.5 ± 13.7 vs. 76.5 ± 12.0 pA, *p* = 0.1411; paired *t* test; *n* = 5, Figure 3F). To determine if adenosine acts directly on the glutamatergic and GABAergic terminals, we recorded the adenosine effect on mEPSCs and mIPSCs. Adenosine did not affect the mEPSCs of small *I*_h_ GABAergic neurons (frequency: 2.5 ± 0.9 vs. 1.5 ± 0.5 Hz in adenosine, *p* = 0.0707; amplitude: 16.7 ± 1.2 vs. 15.8 ± 1.0 pA in adenosine, *p* = 0.0809, paired *t* test; *n* = 6, Figure 3G), or the baseline current of the neurons (−135.8 ± 59.4 vs. −124.7 ± 60.1 pA, *p* = 0.0872, paired *t* test). Adenosine also did not change the frequency or amplitude of mIPSCs in small *I*_h_ GABAergic neurons (frequency: 1.7 ± 0.3 vs. 1.6 ± 0.4 Hz, *p* = 0.8594; amplitude: 24.1 ± 4.4 vs. 25.6 ± 5.8 pA, *p* = 0.4378, paired *t* test; *n* = 4, Figure 3H). These data suggested that adenosine inhibited the action potentials of local glutamatergic and GABAergic neurons but had no effect on glutamate or GABA release from the presynaptic terminals.

### Adenosine decreased the frequency of sEPSCs in PV neurons

Many BF cortically projecting GABAergic neurons are considered as PV positive (Gritti et al., 2003; Furuta et al., 2004). However, only 6.7% of all BF GABAergic neurons and about one quarter of large sized GABAergic neurons are PV positive, even though most PV neurons (∼67%) in BF are GABAergic (McKenna et al., 2013). To determine if adenosine also inhibits the excitatory inputs to PV neurons, we tested the adenosine-induced changes of sEPSCs in a small group of identified PV neurons in MCPO/HDB/VP by using a transgenic mouse model with PV neurons expressing a red fluorescent protein (see Materials and Methods). Our data showed that the frequency of sEPSCs was decreased from 13.7 ± 3.5 to 4.9 ± 0.96 Hz by adenosine (62.1 ± 4.9% decrease, *p* = 0.0292, paired *t* test, *n* = 5), suggesting a presynaptic inhibitory effect of adenosine. All sEPSCs were blocked when DNQX and AP5 were applied to the bath, indicating these excitatory inputs were glutamatergic (*n* = 5). There was no significant change in the amplitude of sEPSCs (48.4 ± 6.7 vs. 48.9 ± 9.7 pA, *p* = 0.8985, paired *t* test) or baseline current (−135.1 ± 9.1 vs. −141.0 ± 15.4 pA, *p* = 0.4630, paired *t* test).

## Discussion

In summary, our data in the mouse suggested that: (1) adenosine inhibited the excitatory glutamatergic inputs to BF cholinergic, GABAergic, and PV neurons; (2) adenosine had no direct *postsynaptic* effect on putative cortically projecting BF neurons; (3) adenosine inhibited the local inhibitory GABAergic inputs onto small *I*_h_ GABAergic neurons; and (4) The A1 receptor antagonist, CPT, blocked all adenosine effects. While presynaptic adenosine effects on mouse BF cholinergic and non-cholinergic neurons have previously been reported (Hawryluk et al., 2012), the adenosine effect on identified BF GABAergic and PV neurons has not been shown before. To the best of our knowledge, this is the first comprehensive work to investigate the adenosine presynaptic and postsynaptic effects on major known cortically projecting BF neurons with identified neurotransmitter/calcium-binding protein phenotypes.

### Sources of glutamatergic inputs to BF neurons

The caudal BF region where we recorded receives glutamatergic inputs from multiple brain regions, including orbital/prefrontal/olfactory cortex, hippocampus, amygdala, lateral septum, hypothalamus, brainstem, and magnocellular BF itself (Carnes et al., 1990). Our data showed that the frequency of sEPSCs was significantly larger than that of mEPSCs, suggesting some of the glutamate release was due to the action potential firing of local glutamatergic neurons. Our data also showed that glutamatergic inputs to cholinergic neurons were inhibited by adenosine in the presence of TTX while those to GABAergic neurons were not, suggesting BF cholinergic and GABAergic/PV neurons receive partially different glutamatergic inputs. Anatomical tract-tracing combined with pre- and postembedding immunocytochemistry experiments have shown that the glutamatergic inputs from prefrontal cortex form synapses with BF GABAergic/PV neurons in HDB/VP/SI but not with cholinergic neurons (Zaborszky et al., 1997). Therefore our data showing a lack of an adenosine effect on mEPSCs of GABAergic neurons suggest that adenosine does not have any effect on the axon terminals of those prefrontal cortex inputs, unless cut cortical axons in the slice are capable of generating action potentials and thereby contribute to the sEPSCs.

### Sources of adenosine-sensitive GABAergic inputs to BF neurons

The areas sending GABAergic afferent to BF have been less studied than those sending glutamatergic afferents. The BF, including the medial septal area and more caudal areas receives GABAergic inputs from hippocampus, amygdala as well as from BF itself (Jones, 2004; McDonald et al., 2012). On brain slices, we found that adenosine had no effect on the inhibitory inputs to cortically projecting BF cholinergic neurons and large *I*_h_ GABAergic neurons, but inhibited the local GABAergic inputs to small *I*_h_ GABAergic neurons. There are a large number of small-to-medium sized GABAergic neurons in the BF, some of which are spontaneously active (McKenna et al., 2013). Thus, the GABAergic neurons which are directly inhibited by adenosine could be located in BF itself. Moreover, the difference in adenosine effect on sIPSCs for different groups of GABAergic neurons, suggested that the large *I*_h_ and small *I*_h_ GABAergic neurons may have different physiological roles and support our separation of these groups based on *I*_h_ characteristics (McKenna et al., 2013).

### Adenosine modulation of BF cholinergic neurons

A recent *in vitro* electrophysiology study showed that adenosine decreased the frequency of sEPSCs and mEPSCs by 46 and 35% respectively in BF cholinergic neurons via A1 receptors (Hawryluk et al., 2012). Our data are broadly consistent with these findings (about 60 and 40% decrease in frequency of sEPSCs and mEPSCs). We did not observe any current or voltage response to adenosine in the presence of TTX, suggesting that adenosine only had a presynaptic inhibition on cholinergic neurons. This finding contrasts previous work in rats where a postsynaptic inhibition of adenosine was reported for about 80% of BF cholinergic neurons (Arrigoni et al., 2006). We think this difference could be due to a species difference or the following technical differences: (1) our tests of postsynaptic modulation were carried out in the presence of TTX while the previous work observed an adenosine-induced hyperpolarization in regular ACSF (Figure 2 in Arrigoni et al., 2006). In fact, we also observed adenosine-induced outward currents while recording sEPSCs in the absence of TTX. In the absence of TTX, action potentials in spontaneously active neurons in the slice can cause release of glutamate, thereby depolarizing the recorded neuron. Inhibition of action potentials by adenosine due to hyperpolarization of the spontaneously active *presynaptic* glutamate neurons can thus affect the *postsynaptic* membrane potential in the cholinergic or GABAergic neurons; (2) Although unlikely, it is possible that all the cholinergic neurons we recorded belong to the minority of cholinergic neurons (20%) reported in the previous study which did not show postsynaptic response to adenosine. We also note that, despite the fact that we did not observe a direct postsynaptic hyperpolarization/outward current, the previously reported adenosine-mediated postsynaptic changes in calcium signaling (Basheer et al., 2002) may still play an important functional role in subtle changes in firing pattern (i.e., burst firing) or neurotransmitter release from these neurons, for instance by activating calcium-dependent potassium conductance responsible for medium and long-duration afterhyperpolarizations.

### Adenosine modulation of BF GABAergic/PV neurons

It has been reported in rats that adenosine may hyperpolarize ∼80% of BF non-cholinergic neurons with large *I*_h_ most likely by inhibiting the hyperpolarization-activated cyclic nucleotide-gated channels (H-channels) (Arrigoni et al., 2006). Even though we confirmed that adenosine inhibits *I*_h_ current in large *I*_h_ neurons (data not shown), we did not observe any measurable current in response to adenosine in the presence of TTX. There are two explanations. First, the different findings on postsynaptic voltage/current response to adenosine could be due to the biased selection of non-cholinergic/GABAergic neurons. In our study, we selected putatively cortically projecting BF GABAergic neurons based on expression of GFP fluorescence, size and electrophysiology (*I*_h_ amplitude), while in the work from Arrigoni et al., the selection was based on the absence of a cholinergic marker, choline acetyltransferase enzyme, and the amplitude of *I*_h_. Thus, it is possible that the two populations may not be identical. A second, more likely explanation is that the H-current only has a small contribution to the resting membrane potential (∼4 mV; Arrigoni et al., 2006; McKenna et al., 2013) and adenosine only has a slight inhibition at potentials close to the resting membrane potential. Thus, even though adenosine inhibits H-channels, this inhibition may not lead to a measurable voltage response. Indeed, as Arrigoni et al. (2006) reported (and we have confirmed), adenosine only has marked effects on H-current at potentials significantly more negative than the resting membrane potential.

### Adenosine inhibition of BF neurons is mediated by A1 receptors

Inhibitory effects of adenosine on wake-promoting neurons are normally mediated by adenosine A1 receptors (Brown et al., 2012). Similarly, our findings here and those of Hawryluk et al. (2012) suggest that all inhibitory effects of adenosine on BF cholinergic and GABAergic neurons are mediated via A1 receptors. These findings are also consistent with *in vivo* studies from our group which showed that inhibiting BF A1 receptors by local perfusion of antisense oligonucleotides directed against the A1 receptor or perfusion of an A1 receptor antagonist blocks the effect of elevated endogenous or exogenous adenosine on sleep and wakefulness (Thakkar et al., 2003; Christie et al., 2008). Taken together, these data support the hypothesis that BF adenosine plays its role in regulating sleep-wakefulness by acting via A1 receptor-mediated inhibition on BF wake-active neurons.

### Physiological significance of adenosine inhibition of BF neurons

**Figure 4:**
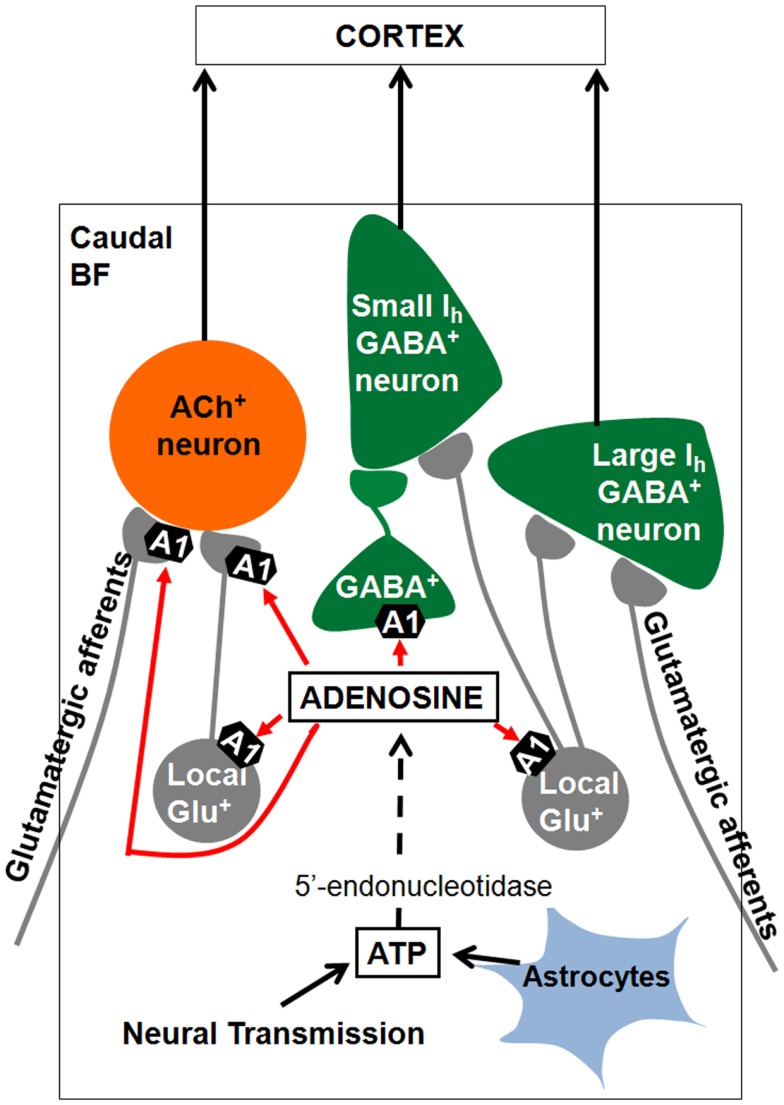
**Summary of results and model of how adenosine may promote sleep in the basal forebrain (BF)**. BF cholinergic and GABAergic/PV neurons project to the cortex and promote cortical activation through their effects on cortical pyramidal neurons and GABAergic interneurons. During prolonged wakefulness extracellular adenosine levels rise due to transport of adenosine by plasma membrane transporters (not shown) or due to degradation of ATP released from glia or as a co-transmitter. Adenosine inhibits cortically projecting BF cholinergic and GABAergic neurons by inhibiting their local glutamatergic inputs through activation of A1 receptors, thus promoting sleep. Adenosine also inhibits the glutamate release from the axon terminals to cholinergic neurons, which could be from either local or distant glutamatergic neurons. Some local GABAergic neurons sending projections to small *I*_h_ GABAergic neurons are also sensitive to adenosine. ACh^+^, cholinergic ATP, adenosine triphosphate.

Our model of how adenosine promotes sleep in the BF is presented in Figure 4. Extracellular adenosine levels rise in the BF during spontaneous wakefulness or during sleep deprivation due to release of adenosine from neurons via transporters or due to degradation of *adenosine triphosphate* (ATP) released from neurons or astrocytes (Kalinchuk et al., 2008; Halassa et al., 2009; Hawryluk et al., 2012; Lovatt et al., 2012). Both *in vivo* and *in vitro* applications of glutamate or glutamate agonists increases extracellular adenosine levels in caudal BF (Wigren et al., 2007; Sims et al., 2013). These data suggest that glutamatergic efferents to BF are involved in BF adenosine production. Our study suggested that the elevated extracellular adenosine in BF inhibits BF putative wake-active cholinergic and GABAergic/PV neurons by inhibiting local glutamate release by activating A1 receptors. The adenosine inhibition of BF cholinergic and glutamatergic neurons would in turn negatively regulates adenosine release itself. As we mentioned earlier, the majority of BF cholinergic and a subset of BF GABAergic neurons (mostly PV) directly project to the neocortex. Thus, adenosine may promote sleep by inhibiting the glutamate release onto these cortically projecting BF neurons. Conversely, the adenosine receptor antagonists, caffeine and theophylline may promote wakefulness, at least in part, by inhibiting the A1 receptors on cortically projecting BF cholinergic and GABAergic/PV neurons.

Previous studies have shown that adenosine A2a receptors in the basal ganglia are required for increases in motor activity and the duration of wakefulness by caffeine (Lindskog et al., 2002; Huang et al., 2005; Lazarus et al., 2011). BF cholinergic neurons display their maximal firing rates during waking or prolonged wakefulness (Manns et al., 2000a) and may enhance cortical theta or gamma activity during active waking or REM sleep (Sarter and Bruno, 2000; Brown et al., 2012). Cortically projecting BF GABAergic/PV neurons are likely to have similar function as BF cholinergic neurons in sleep-wakefulness (Hassani et al., 2009) and may promote cortical activation by modulating gamma activity (Manns et al., 2000b; Kim et al., 2011). Thus, we propose that blockade of A1 receptors on cholinergic and GABA/PV neurons in the BF (shown here) promotes attentive wakefulness (Christie et al., 2008) by blunting sleep homeostasis mechanisms (Thakkar et al., 2003) and by facilitating the high-frequency theta and gamma oscillations in the cortex required for attention (Fries et al., 2001) and cognition (Lisman, 2010).

## Conflict of Interest Statement

The authors declare that the research was conducted in the absence of any commercial or financial relationships that could be construed as a potential conflict of interest.

## References

[B1] Arrigoni E. Chamberlin N. L. Saper C. B. McCarley R. W. (2006). Adenosine inhibits basal forebrain cholinergic and noncholinergic neurons in vitro. Neuroscience 140, 403–413 10.1016/j.neuroscience.2006.02.010 16542780

[B2] Basheer R. Arrigoni E. Thatte H. S. Greene R. W. Ambudkar I. S. McCarley R. W. (2002). Adenosine induces inositol 1,4,5-trisphosphate receptor-mediated mobilization of intracellular calcium stores in basal forebrain cholinergic neurons. J. Neurosci. 22, 7680–7686 12196591 10.1523/JNEUROSCI.22-17-07680.2002 PMC6758010

[B3] Basheer R. Bauer A. Elmenhorst D. Ramesh V. McCarley R. W. (2007). Sleep deprivation upregulates A1 adenosine receptors in the rat basal forebrain. Neuroreport 18, 1895–1899 10.1097/WNR.0b013e3282f262f6 18007182

[B4] Basheer R. Halldner L. Alanko L. McCarley R. W. Fredholm B. B. Porkka-Heiskanen T. (2001). Opposite changes in adenosine A1 and A2A receptor mRNA in the rat following sleep deprivation. Neuroreport 12, 1577–1580 10.1097/00001756-200106130-00013 11409719

[B5] Basheer R. Porkka-Heiskanen T. Stenberg D. McCarley R. W. (1999). Adenosine and behavioral state control: adenosine increases c-Fos protein and AP1 binding in basal forebrain of rats. Brain Res. Mol. Brain Res. 72, 1–10 10.1016/S0169-328X(99)00219-3 10581392

[B6] Basheer R. Strecker R. E. Thakkar M. M. McCarley R. W. (2004). Adenosine and sleep-wake regulation. Prog. Neurobiol. 73, 379–396 10.1016/j.pneurobio.2004.06.004 15313333

[B7] Berntson G. G. Shafi R. Sarter M. (2002). Specific contributions of the basal forebrain corticopetal cholinergic system to electroencephalographic activity and sleep/waking behaviour. Eur. J. Neurosci. 16, 2453–2461 10.1046/j.1460-9568.2002.02310.x 12492440

[B8] Brown R. E. Basheer R. McKenna J. T. Strecker R. E. McCarley R. W. (2012). Control of sleep and wakefulness. Physiol. Rev. 92, 1087–1187 10.1152/physrev.00032.2011 22811426 PMC3621793

[B9] Carnes K. M. Fuller T. A. Price J. L. (1990). Sources of presumptive glutamatergic/aspartatergic afferents to the magnocellular basal forebrain in the rat. J. Comp. Neurol. 302, 824–852 10.1002/cne.903020413 1982006

[B10] Chen L. McKenna J. T. Leonard M. Z. Yanagawa Y. McCarley R. W. Brown R. E. (2010). GAD67-GFP knock-in mice have normal sleep-wake patterns and sleep homeostasis. Neuroreport 21, 216–220 10.1097/WNR.0b013e32833655c4 20051926 PMC3201775

[B11] Christie M. A. Bolortuya Y. Chen L. McKenna J. T. McCarley R. W. Strecker R. E. (2008). Microdialysis elevation of adenosine in the basal forebrain produces vigilance impairments in the rat psychomotor vigilance task. Sleep 31, 1393–1398 18853936 PMC2572744

[B12] Collin T. Chat M. Lucas M. G. Moreno H. Racay P. Schwaller B. (2005). Developmental changes in parvalbumin regulate presynaptic Ca2+ signaling. J. Neurosci. 25, 96–107 10.1523/JNEUROSCI.3748-04.2005 15634771 PMC6725212

[B13] de Lecea L. del Río J. A. Soriano E. (1995). Developmental expression of parvalbumin mRNA in the cerebral cortex and hippocampus of the rat. Brain Res. Mol. Brain Res. 32, 1–13 10.1016/0169-328X(95)00056-X 7494447

[B14] Flagmeyer I. Haas H. L. Stevens D. R. (1997). Adenosine A1 receptor-mediated depression of corticostriatal and thalamostriatal glutamatergic synaptic potentials in vitro. Brain Res. 778, 178–185 10.1016/S0006-8993(97)01060-3 9462890

[B15] Franklin K. B. Paxinos G. (2008). The Mouse Brain in Stereotaxic Coordinates. New York: Academic Press

[B16] Fredholm B. B. (2010). Adenosine receptors as drug targets. Exp. Cell Res. 316, 1284–1288 10.1016/j.yexcr.2010.02.004 20153317 PMC2866745

[B17] Fries P. Reynolds J. H. Rorie A. E. Desimone R. (2001). Modulation of oscillatory neuronal synchronization by selective visual attention. Science 291, 1560–1563 10.1126/science.1055465 11222864

[B18] Furuta T. Koyano K. Tomioka R. Yanagawa Y. Kaneko T. (2004). GABAergic basal forebrain neurons that express receptor for neurokinin B and send axons to the cerebral cortex. J. Comp. Neurol. 473, 43–58 10.1002/cne.20087 15067717

[B19] Greene R. Haas H. (1991). The electrophysiology of adenosine in the mammalian central nervous system. Prog. Neurobiol. 36, 329–341 10.1016/0301-0082(91)90005-L 1678539

[B20] Gritti I. Henny P. Galloni F. Mainville L. Mariotti M. Jones B. E. (2006). Stereological estimates of the basal forebrain cell population in the rat, including neurons containing choline acetyltransferase, glutamic acid decarboxylase or phosphate-activated glutaminase and colocalizing vesicular glutamate transporters. Neuroscience 143, 1051–1064 10.1016/j.neuroscience.2006.09.024 17084984 PMC1831828

[B21] Gritti I. Mainville L. Jones B. E. (1993). Codistribution of GABA- with acetylcholine-synthesizing neurons in the basal forebrain of the rat. J. Comp. Neurol. 329, 438–457 10.1002/cne.903290403 8454735

[B22] Gritti I. Manns I. D. Mainville L. Jones B. E. (2003). Parvalbumin, calbindin, or calretinin in cortically projecting and GABAergic, cholinergic, or glutamatergic basal forebrain neurons of the rat. J. Comp. Neurol. 458, 11–31 10.1002/cne.10505 12577320

[B23] Halassa M. M. Florian C. Fellin T. Munoz J. R. Lee S. Y. Abel T. (2009). Astrocytic modulation of sleep homeostasis and cognitive consequences of sleep loss. Neuron 61, 213–219 10.1016/j.neuron.2008.11.024 19186164 PMC2673052

[B24] Hassani O. K. Lee M. G. Henny P. Jones B. E. (2009). Discharge profiles of identified GABAergic in comparison to cholinergic and putative glutamatergic basal forebrain neurons across the sleep-wake cycle. J. Neurosci. 29, 11828–11840 10.1523/JNEUROSCI.1259-09.2009 19776269 PMC2790860

[B25] Hawryluk J. M. Ferrari L. L. Keating S. A. Arrigoni E. (2012). Adenosine inhibits glutamatergic input to basal forebrain cholinergic neurons. J. Neurophysiol. 107, 2769–2781 10.1152/jn.00528.2011 22357797 PMC3362278

[B26] Henny P. Jones B. E. (2008). Projections from basal forebrain to prefrontal cortex comprise cholinergic, GABAergic and glutamatergic inputs to pyramidal cells or interneurons. Eur. J. Neurosci. 27, 654–670 10.1111/j.1460-9568.2008.06029.x 18279318 PMC2426826

[B27] Huang Z. L. Qu W.-M. Eguchi N. Chen J.-F. Schwarzschild A. Fredholm B. B. (2005). Adenosine A2A, but not A1 receptors mediate the arousal effect of caffeine. Nat. Neurosci. 8, 858–859 10.1038/nn1491 15965471

[B28] Jones B. E. (2004). Activity, modulation and role of basal forebrain cholinergic neurons innervating the cerebral cortex. Prog. Brain Res. 145, 157–169 10.1016/S0079-6123(03)45011-5 14650914

[B29] Kalinchuk A. V. McCarley R. W. Porkka-Heiskanen T. Basheer R. (2011). The time course of adenosine, nitric oxide (NO) and inducible NO synthase changes in the brain with sleep loss and their role in the non-rapid eye movement sleep homeostatic cascade. J. Neurochem. 116, 260–272 10.1111/j.1471-4159.2010.07100.x 21062286 PMC3042163

[B30] Kalinchuk A. V. McCarley R. W. Stenberg D. Porkka-Heiskanen T. Basheer R. (2008). The role of cholinergic basal forebrain neurons in adenosine-mediated homeostatic control of sleep: lessons from 192 IgG-saporin lesions. Neuroscience 157, 238–253 10.1016/j.neuroscience.2008.08.040 18805464 PMC3678094

[B31] Kim T. McKenna J. T. McNally J. M. Winston S. Yang C. Chen L. (2011). Optogenetic stimulation of parvalbumin-positive basal forebrain neurons entrains cortical gamma oscillations and promotes wakefulness. Soc. Neurosci. Abs. 286.15.

[B32] Lazarus M. Shen H. Y. Cherasse Y. Qu W.-M. Huang Z.-L. Bass C. E. (2011). Arousal effect of caffeine depends on adenosine A_2A_ receptors in the shell of the nucleus accumbens. J. Neurosci. 31, 10067–10075 10.1523/JNEUROSCI.6730-10.2011 21734299 PMC3153505

[B33] Lindskog M. Svenningsson P. Pozzi L. Kim Y. Flenberg A. A. Bibb J. A. (2002). Involvement of DARPP-32 phosphorylation in the stimulant action of caffeine. Nature 418, 774–778 12181566 10.1038/nature00817

[B34] Lisman J. (2010). Working memory: the importance of theta and gamma oscillations. Curr. Biol. 20, R490–R492 10.1016/j.cub.2010.04.011 20541499

[B35] Lohmann C. Friauf E. (1996). Distribution of the calcium-binding proteins parvalbumin and calretinin in the auditory brainstem of adult and developing rats. J. Comp. Neurol. 367, 90–109 10.1002/(SICI)1096-9861(19960325)367:1<90::AIDCNE7>;3.0.CO;2-E 8867285

[B36] Lovatt D. Xu Q. Liu W. Takano T. Smith N. Schnermann J. (2012). Neuronal adenosine release, and not astrocytic ATP release, mediates feedback inhibition of excitatory activity. Proc. Natl. Acad. Sci. U.S.A. 109, 6265–6270 10.1073/pnas.1120997109 22421436 PMC3341061

[B37] Manns I. D. Alonso A. Jones B. E. (2000a). Discharge properties of juxtacellularly labeled and immunohistochemically identified cholinergic basal forebrain neurons recorded in association with the electroencephalogram in anesthetized rats. J. Neurosci. 20, 1505–1518 10662840 10.1523/JNEUROSCI.20-04-01505.2000 PMC6772366

[B38] Manns I. D. Alonso A. Jones B. E. (2000b). Discharge profiles of juxtacellularly labeled and immunohistochemically identified GABAergic basal forebrain neurons recorded in association with the electroencephalogram in anesthetized rats. J. Neurosci. 20, 9252–9263 11125003 10.1523/JNEUROSCI.20-24-09252.2000 PMC6773015

[B39] McDonald A. J. Mascagni F. Zaric V. (2012). Subpopulations of somatostatin-immunoreactive non-pyramidal neurons in the amygdala and adjacent external capsule project to the basal forebrain: evidence for the existence of GABAergic projection neurons in the cortical nuclei and basolateral nuclear complex. Front. Neural Circuits 6:46 10.3389/fncir.2012.00046 22837739 PMC3402756

[B40] McKenna J. T. Yang C. Franciosi S. Winston S. Abarr K. K. Rigby M. S. (2013). Distribution and intrinsic membrane properties of basal forebrain GABAergic and parvalbumin neurons in the mouse. J. Comp. Neurol. 521, 1225–1250 10.1002/cne.23290 23254904 PMC3627393

[B41] McNally J. M. McCarley R. W. McKenna J. T. Yanagawa Y. Brown R. E. (2011). Complex receptor mediation of acute ketamine application on in vitro gamma oscillations in mouse prefrontal cortex: modeling gamma band oscillation abnormalities in schizophrenia. Neuroscience 199, 51–63 10.1016/j.neuroscience.2011.10.015 22027237 PMC3237956

[B42] Moruzzi G. Magoun H. W. (1949). Brain stem reticular formation and activation of the EEG. Electroencephalogr. Clin. Neurophysiol. 1, 455–473 10.1016/0013-4694(49)90219-9 18421835

[B43] Pomeranz J. L. Munsell C. R. Harris J. L. (2013). Energy drinks: an emerging public health hazard for youth. J. Public Health Policy 34, 254–271 10.1057/jphp.2013.6 23486464

[B44] Porkka-Heiskanen T. Strecker R. E. McCarley R. W. (2000). Brain site-specificity of extracellular adenosine concentration changes during sleep deprivation and spontaneous sleep: an in vivo microdialysis study. Neuroscience 99, 507–517 10.1016/S0306-4522(00)00220-7 11029542

[B45] Porkka-Heiskanen T. Strecker R. E. Thakkar M. Bjorkum A. A. Greene R. W. McCarley R. W. (1997). Adenosine: a mediator of the sleep-inducing effects of prolonged wakefulness. Science 276, 1265–1268 10.1126/science.276.5316.1265 9157887 PMC3599777

[B46] Rye D. B. Wainer B. H. Mesulam M. M. Mufson E. J. Saper C. B. (1984). Cortical projections arising from the basal forebrain: a study of cholinergic and noncholinergic components employing combined retrograde tracing and immunohistochemical localization of choline acetyltransferase. Neuroscience 13, 627–643 10.1016/0306-4522(84)90083-6 6527769

[B47] Sarter M. Bruno J. P. (2000). Cortical cholinergic inputs mediating arousal, attentional processing and dreaming: differential afferent regulation of the basal forebrain by telencephalic and brainstem afferents. Neuroscience 95, 933–952 10.1016/S0306-4522(99)00487-X 10682701

[B48] Semba K. (2000). Multiple output pathways of the basal forebrain: organization, chemical heterogeneity, and roles in vigilance. Behav. Brain Res. 115, 117–141 10.1016/S0166-4328(00)00254-0 11000416

[B49] Sims R. E. Wu H. H. Dale N. (2013). Sleep-wake sensitive mechanisms of adenosine release in the basal forebrain of rodents: an in vitro study. PLoS One 8:e53814 10.1371/journal.pone.0053814 23326515 PMC3543262

[B50] Sun H. W. Qiao F. X. Liu G. Y. (2006). Characteristic of theophylline imprinted monolithic column and its application for determination of xanthine derivatives caffeine and theophylline in green tea. J. Chromatogr. A 1134, 194–200 10.1016/j.chroma.2006.09.004 17046776

[B51] Thakkar M. M. Winston S. McCarley R. W. (2003). A1 receptor and adenosinergic homeostatic regulation of sleep-wakefulness: effects of antisense to the A1 receptor in the cholinergic basal forebrain. J. Neurosci. 23, 4278–4287 12764116 10.1523/JNEUROSCI.23-10-04278.2003 PMC2002519

[B52] Wigren H. K. Schepens M. Matto V. Stenberg D. Porkka-Heiskanen T. (2007). Glutamatergic stimulation of the basal forebrain elevates extracellular adenosine and increases the subsequent sleep. Neuroscience 147, 811–823 10.1016/j.neuroscience.2007.04.046 17574765

[B53] Yang C. Franciosi S. McCarley R. W. Yanagawa Y. Basheer R. Brown R. E. (2011). Adenosine and adenosine triphosphate act directly and indirectly to decrease the excitability of cholinergic and GABAergic basal forebrain neurons in the mouse. Soc. Neurosci. Abs. 397.16.

[B54] Zaborszky L. Gaykema R. P. Swanson D. J. Cullinan W. E. (1997). Cortical input to the basal forebrain. Neuroscience 79, 1051–1078 10.1016/S0306-4522(97)00049-3 9219967

